# Synthesis, Structures, and Magnetism of Four One-Dimensional Complexes Using [Ni(CN)_4_]^2−^ and Macrocyclic Metal Complexes

**DOI:** 10.3390/molecules28114529

**Published:** 2023-06-02

**Authors:** Guangchuan Ou, Qiong Wang, Yingzhi Tan, Qiang Zhou

**Affiliations:** College of Chemistry and Bioengineering, Hunan University of Science and Engineering, Yongzhou 425199, China

**Keywords:** macrocyclic metal complexes, tetracyanonicolate, magnetism

## Abstract

Four one-dimensional complexes, denoted as [NiL_1_][Ni(CN)_4_] (**1**), [CuL_1_][Ni(CN)_4_] (**2**), [NiL_2_][Ni(CN)_4_]·2H_2_O (**3**), and [CuL_2_][Ni(CN)_4_]·2H_2_O (**4**) (L_1_ = 1,8-dimethyl-1,3,6,8,10,13-hexaaza-cyclotetradecane; L_2_ = 1,8-dipropyl-1,3,6,8,10,13-hexaazacyclotetradecane) were synthesized by reacting nickel/copper macrocyclic complexes with K_2_[Ni(CN)_4_]. Subsequently, the synthesized complexes were characterized using elemental analysis, infrared spectroscopy analysis, thermogravimetric analysis, and X-ray powder diffraction. Single-crystal structure analysis revealed that the Ni(II)/Cu(II) atoms were coordinated by two nitrogen atoms from [Ni(CN)_4_]^2−^ with four nitrogen atoms from a macrocyclic ligand, forming a six-coordinated octahedral coordination geometry. Nickel/copper macrocyclic complexes were bridged by [Ni(CN)_4_]^2−^ to construct one-dimensional chain structures in **1**–**4**. The characterization results showed that the four complexes obeyed the Curie–Weiss law with a weak antiferromagnetic exchange coupling.

## 1. Introduction

The rational construction of one to three-dimensional porous coordination complexes has attracted considerable attention because of their potential applications in gas adsorption and separation, catalysis, and magnetism [1,2,3,4]. The design and assembly of different dimensional cyano-bridged complexes have been extensively researched in recent decades. Cyano-bridged complexes with superior magnetic properties can be constructed using cyano complexes, such as [Ag(CN)_2_]^−^, [Cu(CN)_3_]^2−^, [M(CN)_4_]^2−^ (M = Cd, Ni, Pd, and Pt), and [M(CN)_6_]^3−^ (M = W, Mo, and Fe) [5,6,7,8,9,10,11,12,13,14,15]. Studies on tetracyanometallate anions [Ni(CN)_4_]^2−^ exhibiting bridging characteristics with either one or two of the cyano groups have been reported [16,17,18,19,20,21,22,23,24,25,26,27,28].

**Scheme 1:**
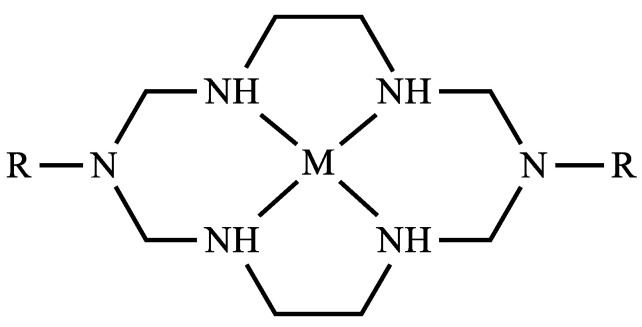
Structural formula of macrocyclic complexes (R_1_ = CH_3_, R_2_ = C_3_H_7_, and M = Ni or Cu).

In previous reports, a few cyano-bridged complexes with one-dimensional helical chains were prepared and structurally characterized using transition metal macrocyclic complexes [NiL_0_]^2+^ (L_0_ = 5,5,7,12,12,14-hexamethyl-1,4,8,11-tetraazacyclo-tetradecane) and [Ni(CN)_4_]^2−^ [24]. As a continuation of the above research, we used different macrocyclic ligands (L_1_ = 1,8-dimethyl-1,3,6,8,10,13-hexaazacyclotetradecane, L_2_ = 1,8-dipropyl-1,3,6,8,10,13-hexaazacyclotetradecane, represented in Scheme 1) for the construction of cyano-bridged complexes. In this study, we synthesized four cyano-bridged complexes: [NiL_1_][Ni(CN)_4_] (**1**), [CuL_1_][Ni(CN)_4_] (**2**), [NiL_2_][Ni(CN)_4_]·2H_2_O (**3**), and [CuL_2_][Ni(CN)_4_]·2H_2_O (**4**), which were isolated from the reactions of [ML](ClO_4_)_2_ with [Ni(CN)_4_]^2−^. In addition, the structures and magnetism of four cyano-bridged complexes were analyzed.

## 2. Results and Discussion

### 2.1. Description of Structures

**Figure 1:**
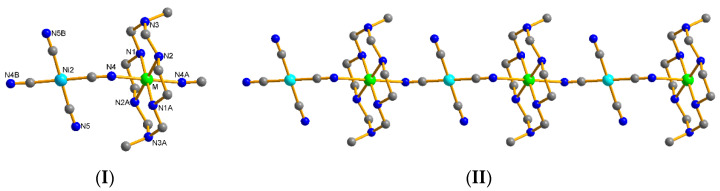
(**I**) The structure of complexes **1** and **2** (M = Ni or Cu) and the symmetry codes for the generated atoms: A(1−*x*, 1−*y*, 1−*z*), B(−*x*, −*y*, 1−*z*); (**II**) The [Ni(CN)_4_]^2−^ bridges macrocyclic complex [ML_1_]^2+^ to form a one-dimensional chain along the *b*-axis.

Figure 1 shows the similarity between the structures of complexes **1** and **2**. One asymmetric unit of the structure of complex **1** contained one [NiL_1_]^2+^ cation and one [Ni(CN)_4_]^2−^ anion. Each Ni(1) was on an inversion center and coordinated with four nitrogen atoms, namely N(1), N(2), N(1A), and N(2A) (symmetry code: A, 1–*x*, 1–*y*, and 1–*z*), of the macrocyclic ligand in the equatorial plane and two nitrogen atoms, namely, N(4) and N(4B) (symmetry code: B, –*x*, –*y*, 1–*z*), of [Ni(CN)_4_]^2−^ in axial positions, forming a six-coordinated octahedral geometry in **1**. The distances [2.0498(16) and 2.0712(16) Å] of Ni(1)-N(macrocycle) were shorter than that of the Ni(1)-N(cyano) [2.1489(17) Å], and they were longer than that of the Ni(2)-C(cyano) [1.858(2)-1.866(2) Å] (Table 1). The [Ni(CN)_4_]^2−^ anion bridged the macrocyclic complex [NiL_1_]^2+^ cation in trans-positions to form a one-dimensional chain along the *b*-axis (Figure 1). One-dimensional linear chains were formed when *trans*-M(II) tetradentate macrocyclic complexes were used because of steric hindrance. Four possible helical isomers can be formed when *cis*-M(II) tetradentate macrocyclic complexes and [Ni(CN)_4_]^2−^ building blocks are used [18,19,24].

**Figure 2:**
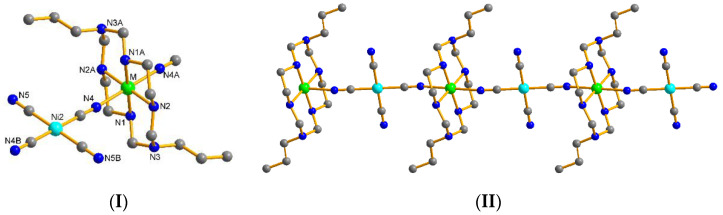
(**I**) The structure of complexes **3** and **4** (M = Ni or Cu) and the symmetry codes for the generated atoms: A(1−*x*, 1−*y*, 1−*z*), B(−*x*, −*y*, 1−*z*); (**II**) The [Ni(CN)_4_]^2−^ bridges macrocyclic complex [ML_2_]^2+^ to form a one-dimensional chain along the *b*-axis.

The six-coordinated Cu(II) ions of complex **2** displayed a distorted octahedral geometry by coordinating with four nitrogen atoms, namely, N(1), N(2), N(1A), and N(2A) (symmetry code: A, 1–*x*, 1–*y*, 1–*z*), of macrocyclic ligand in the equatorial plane and two nitrogen atoms, namely, N(5) and N(5B) (symmetry code: B, –*x*, –*y*, 1–*z*) of [Ni(CN)_4_]^2−^ in axial positions (Figure 1). The Cu(1)-N(macrocycle) distances [2.0009(15) and 2.0240(16) Å] were shorter than the Cu(1)-N(cyano) distances [2.563(2) Å] due to the Jahn-Teller effect, but longer than the distances [1.861(2)-1.865(2) Å] of Ni(1)-C(cyano) (Table 1). The [Ni(CN)_4_]^2−^ anion bridged the macrocyclic complex [CuL_1_]^2+^ cation in *trans*-positions to form a one-dimensional chain along the *b*-axis in **2** (Figure 1). Complexes **3** and **4** exhibited similar one-dimensional structures. The asymmetric unit of complex **3**/**4** contained one [Ni/CuL_2_]^2+^ cation, one [Ni(CN)_4_]^2−^ anion, and two water molecules (Figure 2). The Ni(II)/Cu(II) ions were coordinated by four nitrogen atoms of ligands along the equatorial plane and two nitrogen atoms of [Ni(CN)_4_]^2−^ in axial positions. The [Ni(CN)_4_]^2−^ anion bridged the macrocyclic complex [Ni/CuL_2_]^2+^ cation in *trans*-positions to form a one-dimensional chain along the *b*-axis in **3**/**4** (Figure 2).

Due to the Jahn-Teller effect, the axial Cu–N(cyano) bonds were considerably longer [2.563(2) in **2** and 2.410(2) Å in **4** (Table 1)] than those of the equatorial with mean values of Cu–N(macrocycle) bonds of 2.0125(15) and 2.0159(19) Å in **2** and **4**, respectively. The five-membered intra-chelate N-M-N angles had similar values [85.85(7), 86.28(7), 85.66(10), and 85.93(8) Å for **1**–**4,** respectively]. The Ni–C–N angles slightly deviated from linearity, and the maximum deviation was observed for the Ni–C–N angle [171.6(7)°] in complex **1**, and the Ni–C–N angles of coordinated cyano groups [171.6(7)°, 175.7(9)°, 177.1(2)°, and 178.0(2)° for **1**–**4**, respectively] were smaller than those of uncoordinated cyano groups [176.0(2)°, 177.3(2)°, 178.9(3)°, and 178.8(3)° for **1**–**4,** respectively]. However, the M–C–N angles significantly deviated from linearity. The M–C–N angles of the L_1_ ligand [149.7(5)° and 127.8(8)° for **1** and **2**, respectively] were smaller than the M–C–N angles of L_2_ ligand [168.4(2)° and 160.8(2)° for **3** and **4**, respectively], shortening the distances of neighboring macrocyclic metal center [9.834(2), 9.906(2), 10.187(2), and 10.647(2) Å for **1**–**4**].

### 2.2. IR Spectra

The infrared spectra (Figures S1–S4) of complexes **3** and **4** had broad absorption bands near 3400 cm^–1^, and the absorption was attributed to the stretching vibration of O–H. The N–H absorption bands appeared near 3200 cm^–1^, and the absorption bands at 2136 (s) and 2125 (s), 2123 (s) and 2119 (s), 2152 (s) and 2131 (s), and 2136 (s) and 2129 (s) cm^–1^ were assigned to υ(M–CN–M) and υ(M–CN) (M = Ni or Cu) in complexes **1**–**4**.

### 2.3. XRD and TG

**Figure 3:**
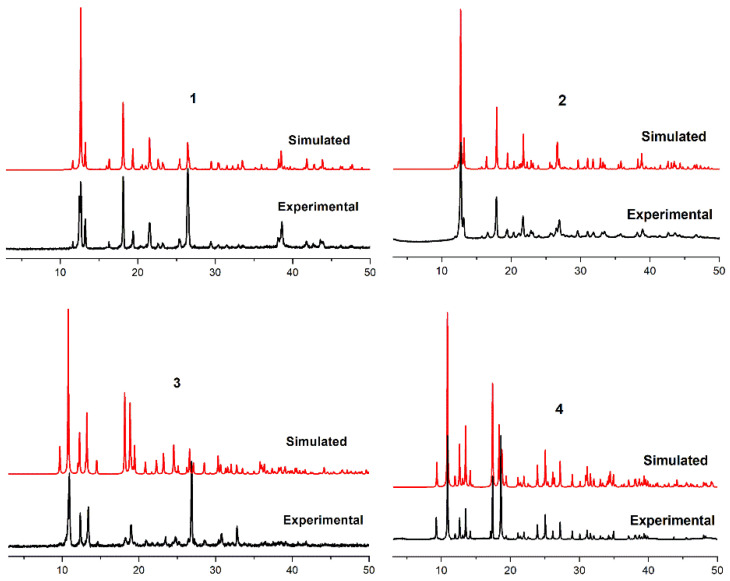
XRD patterns of complexes **1**–**4**.

X-ray powder diffraction measurements for **1**–**4** (Figure 3) showed that the peaks in the measured patterns for both complexes closely matched those in the simulated patterns generated from single-crystal diffraction data, indicating that single phases were formed.

**Figure 4:**
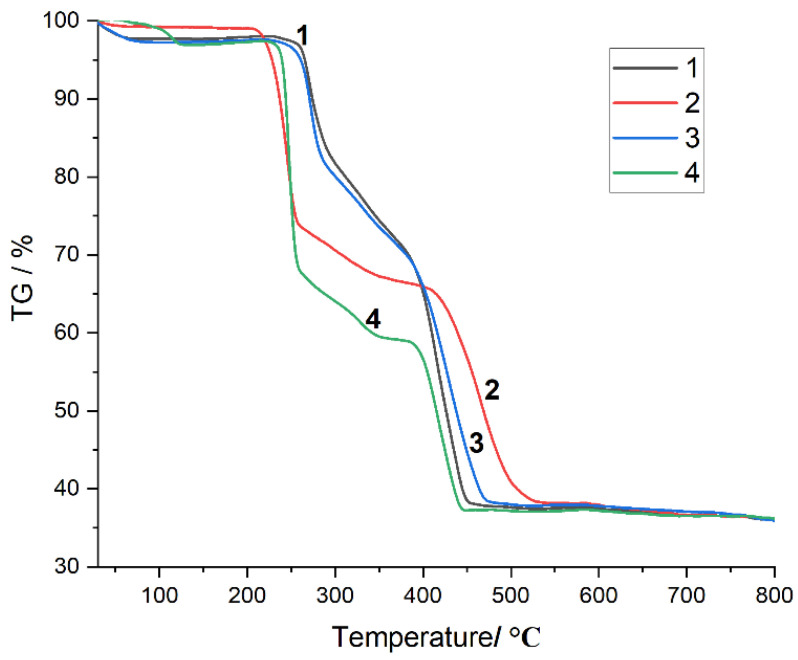
TGA curves of complexes **1**–**4**.

Figure 4 shows the thermogravimetric analysis (TGA) curves of both complexes. The TGA of complex **1** revealed that a weight loss of approximately 24.2% occurred from room temperature to 613 K, corresponding to the release of adsorbed water from the air (2.3%) and four CN^−^ (calcd 22.5%).

The decomposition of the macrocyclic structure was observed after the further heating of the macrocyclic ligand. The TGA curve for **2** showed the first weight loss from room temperature to 523 K, and the observed weight loss of 22.8% was related to the release of four CN^−^ (calcd 22.7%). Then, the macrocyclic structure began to decompose after the macrocyclic ligand was further heated.

The TGA curve for complex **3** revealed that a weight loss of approximately 27.2% occurred from room temperature to 630K, which was attributed to the release of adsorbed water from the air (2.3%), two lattice water molecules (calcd 6.5%), and four CN^−^ (calcd 18.6%). The weight loss was attributed to the release of structural water molecules and four CN^−^. The TGA curve of complex **4** was similar to that of complex **3**, which revealed that an initial weight loss of 25.3% (calcd 25.5%) occurred from room temperature to 496 K, corresponding to the release of structural water molecules and four CN^−^.

### 2.4. Magnetism

**Figure 5:**
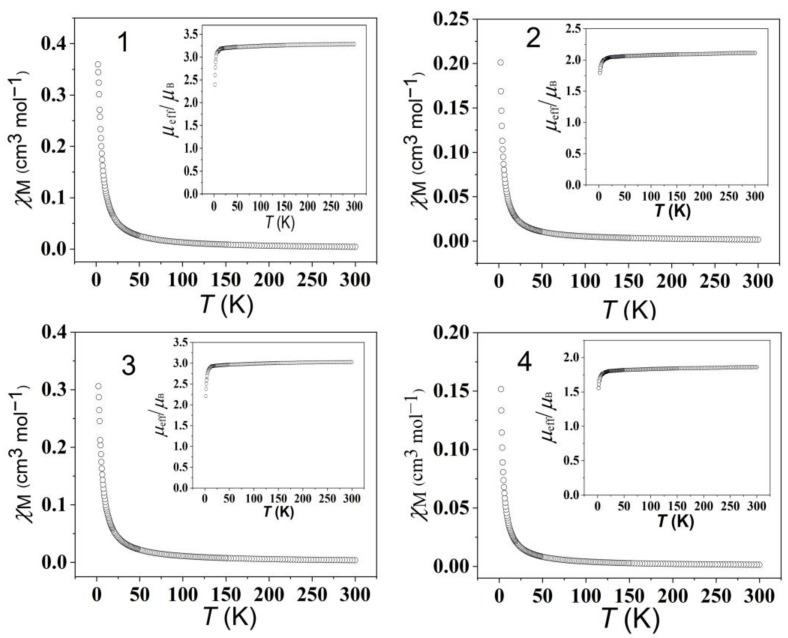
Plots of *χ*_M_ vs. *T* and *μ*_eff_/*μ*_B_ vs. *T* (inset) of complexes **1**–**4** in the temperature range of 2–300 K.

Magnetic susceptibility measurements were performed to investigate the magnetic behaviors of complexes **1**–**4** at 1000 G within the temperature range of 2–300 K. Plots of *χ*_M_ vs. *T* and *µ*_eff_/*µ*_B_ vs. T of the complexes within the temperature range of 2–300 K are shown in Figure 5. Complexes **1** and **3** exhibited similar magnetic properties, and their *µ*_eff_/*µ*_B_ values within the temperature range of 7–300 K were close to the theoretical value expected for two unpaired d electrons in Ni(II) ions. In addition, complexes **2** and **4** exhibited similar magnetic properties, and their *µ*_eff_/*µ*_B_ values within the temperature range of 7–300 K were close to the theoretical value expected for an unpaired d electron in Cu(II) ions.

The magnetic susceptibility measurements between 2 and 300 K yielded *C* = 1.35 cm^3^·K·mol^−1^ and *θ* = −1.85 K for **1**, while the corresponding values were *C* = 0.56 cm^3^·K·mol^−1^ and *θ* = −2.21 K for **2**, *C* = 1.15 cm^3^·K·mol^−1^ and *θ* = −1.79 K for **3**, and *C* = 0.43 cm^3^·K·mol^−1^ and *θ* = −2.20 K for **4**. The antiferromagnetic interactions were confirmed because of the negative Weiss constants *θ* in complexes **1**–**4**. Weak antiferromagnetic interactions were also observed in analogous complexes Cu(cyclam)M(CN)_4_ [cyclam = 1,4,8,11−tetraazacyclotetradecane, M = Ni, Pd], and the reported values of the Weiss constants *θ* were −0.79 K for M = Ni [21]. The characterization results showed that the four complexes **1**–**4** obeyed the Curie–Weiss law with a weak antiferromagnetic exchange coupling.

## 3. Materials and Methods

The Ni(II) and Cu(II) macrocycle complexes were prepared following the previous report procedure [29]. All of the chemicals used in this work were commercially available and were used without further purification. Elemental analyses were carried out using an Elementar Micro Cube elemental analyzer. Infrared spectra were recorded in the 4000−400 cm^−1^ region using KBr pellets and a Bruker EQUINOX 55 spectrometer (Bruker, Germany). Thermogravimetric analyses were performed using a Netzsch STA 449F3 instrument (Netzsch, Germany) in flowing air at a heating rate of 10 °C·min^−1^. X-ray powder diffraction data were recorded using a Bruker D8 ADVANCE X-ray powder diffractometer (Cu Kα radiation, λ = 1.5418 Å, Bruker, Germany). Magnetic susceptibility measurements were conducted to determine the magnetic behaviors of both complexes at 1000 G in a temperature range of 2–300 K.

### Preparation of the Compounds

[NiL_1_][Ni(CN)_4_] (1): An aqueous solution (20 mL) of K_2_[Ni(CN)_4_] (0.024 g, 0.1 mmol) was layered on an acetonitrile solution (20 mL) of NiL_1_(ClO_4_)_2_ (0.048 g, 0.1 mmol). After a few days, green prism crystals of **1** with ~37% yield were obtained. Elemental Anal. Found: C, 37.62; H, 5.65; N, 31.25%. Calcd for C_7_H_13_NiN_5_: C, 37.22; H, 5.80; N, 31.00%. IR (KBr): 3226 (s), 2946 (m), 2870 (m), 2136 (s), 2125 (s), 1435 (m), 1283 (s), 1059 (m), 1015 (m), 834 (m), and 603 (m) cm^–1^.

[CuL_1_][Ni(CN)_4_] (2): An aqueous solution (20 mL) of K_2_[Ni(CN)_4_] (0.024 g, 0.1 mmol) was layered on an acetonitrile solution (20 mL) of CuL_1_(ClO_4_)_2_ (0.049 g, 0.1 mmol). After some weeks, red prism crystals of **2** with ~42% yield were obtained. Elemental Anal. Found: C, 36.45; H, 5.98; N, 30.92%. Calcd for C_14_H_26_CuN_10_Ni: C, 36.82; H, 5.74; N, 30.67%. IR (KBr): 3247 (m), 3180 (m), 2960 (w), 2123 (s), 2119 (s), 1430 (m), 1286 (m), 1090 (m), 1007 (s), 840 (m), 612 (m) cm^–1^.

[NiL_2_][Ni(CN)_4_]·2H_2_O (3): Crystals of complex **3** with ~28% yield were prepared following the similar synthetic method of complex **1**; however, [NiL_2_](ClO_4_)_2_ (0.054 g, 0.1 mmol) was used in place of [NiL_1_](ClO_4_)_2_. Elemental Anal. Found: C, 39.92; H, 7.28; N, 25.52%. Calcd. for C_18_H_38_Ni_2_N_10_O_2_: C, 39.75; H, 7.04; N, 25.75%. IR (KBr): 3590 (m), 3405 (m), 3222 (m), 2870 (w), 2152 (s), 2131 (s), 1430 (w), 1284 (w), 1080 (m), 1019 (s), 908 (m), and 620 (m) cm^–1^.

[CuL_2_][Ni(CN)_4_]·2H_2_O (4): Crystals of complex **4** with ~35% yield were synthesized using a similar synthetic method of complex **1**; however, [CuL_2_](ClO_4_)_2_ (0.055 g, 0.1 mmol) was used in place of [NiL_1_](ClO_4_)_2_. Elemental Anal. Found: C, 39.65; H, 6.65; N, 25.35%. Calcd. for C_18_H_38_CuNiN_10_O_2_: C, 39.39; H, 6.98; N, 25.52%. IR (KBr): 3599 (m), 3372 (m), 3181 (m), 2925 (w), 2136 (s), 2129 (s), 1426 (w), 1285 (w), 1087 (m), 1012 (s), 842 (m), and 627 (m) cm^–1^.

Crystal Structure Determination. Single-crystal data for **1**–**4** were collected using a Bruker Smart Apex II diffractometer (Bruker, Germany) with Mo-Kα radiation (λ = 0.71073 Å). All empirical absorption corrections were applied using the SADABS program [30]. All structures were solved using direct methods, which yielded the positions of all non-hydrogen atoms. The positions were first refined isotropically, then anisotropically. All the hydrogen atoms of the ligands were placed in calculated positions with fixed isotropic thermal parameters and included in the structure factor calculations in the final stage of full-matrix least-squares refinement. All calculations were performed using the SHELXTL 5.1 software package [31]. For complexes **3** and **4**, the hydrogen atoms bonded to oxygen were introduced at idealized positions and refined as riders with isotropic displacement parameters assigned 1.2 times the *U*_eq_ value of the corresponding bonding partner. Selected bond lengths and angles are listed in Table 1. The crystallographic data of **1**–**4** are summarized in Table 2.

## 4. Conclusions

In this work, four one-dimensional linear chains were successfully obtained between the reactions of macrocyclic nickel/copper complexes and [Ni(CN)_4_]^2−^. All complexes exhibited one-dimensional linear chain structures, which were formed by bridging [NiL]^2+^/[CuL]^2+^ with [Ni(CN)_4_]^2−^ moieties. The magnetic susceptibilities revealed Curie-Weiss behavior for complexes **1**–**4** and the existence of weak antiferromagnetic exchange coupling.

## Figures and Tables

**Table 1:** Selected bond distances (Å) and angles (°).

1					
Bond	Length	Bond	Length	Bond	Length
Ni(1)-N(1)	2.0498(16)	Ni(1)-N(2)	2.0712(16)	Ni(1)-N(4)	2.1489(17)
Ni(2)-C(6)	1.866(2)	Ni(2)-C(7)	1.858(2)		
Bond	Angle	Bond	Angle	Bond	Angle
N(1)-Ni(1)-N(2)	94.15(7)	N(1)-Ni(1)-N(2) ^#1^	85.85(7)	N(1)-Ni(1)-N(4) ^#1^	90.27(6)
N(2)-Ni(1)-N(4) ^#1^	88.33(6)	N(1)-Ni(1)-N(4)	89.73(6)	N(2)-Ni(1)-N(4)	91.67(6)
N(5)-C(7)-Ni(2)	176.0(2)	N(4)-C(6)-Ni(2)	171.6(7)	C(6)-N(6)-Ni(1)	149.7(5)
2					
Bond	Length	Bond	Length	Bond	Length
Cu(1)-N(1)	2.0009(15)	Cu(1)-N(2)	2.0240(16)	Cu(1)-N(5)	2.563(2)
Ni(1)-C(7)	1.865(2)	Ni(1)-C(6)	1.861(2)		
Bond	Angle	Bond	Angle	Bond	Angle
N(1)-Cu(1)-N(2)	93.72(7)	N(1)-Cu(1)-N(2) ^#1^	86.28(7)	C(6)-Ni(1)-C(7)	88.08(9)
C(6)-Ni(1)-C(7) ^#2^	91.92(9)	N(5)-C(7)-Ni(1)	175.7(9)	N(4)-C(6)-Ni(1)	177.3(2)
C(7)-N(5)-Cu(1)	127.8(8)				
3					
Bond	Length	Bond	Length	Bond	Length
Ni(1)-N(1)	2.061(2)	Ni(1)-N(2)	2.068(2)	Ni(1)-N(4)	2.118(2)
Ni(2)-C(8)	1.864(3)	Ni(2)-C(9)	1.862(3)		
Bond	Angle	Bond	Angle	Bond	Angle
N(1)-Ni(1)-N(2) ^#1^	85.66(10)	N(1)-Ni(1)-N(2)	94.34(10)	N(1)-Ni(1)-N(4)	92.09(10)
N(2)-Ni(1)-N(4)	91.21(9)	N(1)-Ni(1)-N(4) ^#1^	87.91(10)	N(2)-Ni(1)-N(4) ^#1^	88.79(9)
C(9)-Ni(2)-C(8)	91.14(11)	C(9)-Ni(2)-C(8) ^#3^	88.86(11)	N(4)-C(8)-Ni(2)	177.1(2)
N(5)-C(9)-Ni(2)	178.9(3)	C(8)-N(4)-Ni(1)	148.4(2)		
4					
Bond	Length	Bond	Length	Bond	Length
Cu(1)-N(1)	2.016(2)	Cu(1)-N(2)	2.0159(19)	Cu(1)-N(4)	2.410(2)
Ni(1)-C(8)	1.864(3)	Ni(1)-C(9)	1.865(3)		
Bond	Angle	Bond	Angle	Bond	Angle
N(1)-Cu(1)-N(2) ^#4^	85.93(8)	N(1)-Cu(1)-N(2)	94.07(8)	N(1)-Cu(1)-N(4) ^#4^	91.93(8)
N(2)-Cu(1)-N(4) ^#4^	91.37(9)	N(1)-Cu(1)-N(4)	88.07(8)	N(2)-Cu(1)-N(4)	88.63(9)
C(8)-Ni(1)-C(9)	90.18(11)	C(8)-Ni(1)-C(9) ^#5^	89.82(11)	N(4)-C(8)-Ni(1)	178.0(2)
N(5)-C(9)-Ni(1)	178.8(3)	C(8)-N(4)-Cu(1)	160.8(2)		

Symmetry transformation: ^#1^ −*x* + 1, −*y* + 1, −*z* + 1; ^#2^ −*x*, −*y* + 1, −*z* + 1; ^#3^ −*x* + 2, −*y* + 1, −*z*; ^#4^ −*x* + 1, −*y* + 1, −*z* + 2; ^#5^ −*x* + 2, −*y* + 1, −*z* + 1.

**Table 2:** Crystallographic data.

Compound	[NiL_1_][Ni(CN)_4_]	[CuL_1_][Ni(CN)_4_]	[NiL_2_][Ni(CN)_4_]·2H_2_O	[CuL_2_][Ni(CN)_4_]·2H_2_O
Empirical formula	C_14_H_26_Ni_2_N_10_	C_14_H_26_CuNiN_10_	C_18_H_38_Ni_2_O_2_N_10_	C_18_H_38_CuNiN_10_O_2_
Formula weight	451.86	456.70	544.00	548.83
Temperature (K)	296(2)	296(2)	296(2)	296(2)
Crystal system	Monoclinic	Monoclinic	Triclinic	Triclinic
Space group	*P*2(1)/*n*	*P*2(1)/*n*	*P*-1	*P*-1
*a*/Å	9.834(3)	9.906(4)	8.588(8)	8.400(4)
*b*/Å	9.167(3)	9.110(4)	8.873(8)	8.771(4)
*c*/Å	10.910(4)	10.785(5)	10.001(9)	10.098(4)
*α*/°	90	90	80.930(11)	81.180(4)
*β*/°	95.177(4)	92.268(4)	65.954(10)	69.590(4)
*γ*/°	90	90	67.544(10)	67.991(4)
*V*/Å^3^	979.5(6)	972.6(7)	643.1(10)	646.2(5)
*Z*	4	2	2	1
*D*_c_/Mg cm^−3^	1.532	1.559	1.405	1.410
*μ*/mm^−1^	1.943	2.082	1.498	1.585
*F* (000)	472	474	288	289
Crystal size (mm)	0.36 × 0.28 × 0.23	0.46 × 0.36 × 0.28	0.28 × 0.23 × 0.15	0.38 × 0.26 × 0.20
*θ* range	2.67–27.46	2.74–27.47	2.23–27.44	2.15–27.53
Collected/unique	10687/2221(0.022)	10594/2206(0.021)	7227/2838(0.0295)	6873/2872(0.0306)
Completeness to *θ*	99.3%	99.1%	97.0%	96.3%
Goodness-of-fit on *F*^2^	1.041	1.012	0.979	0.991
Final *R* indices [*I* > 2*σ* (*I*)]	0.0279, 0.0721	0.0268, 0.0716	0.0340, 0.0828	0.0356, 0.0893
*R* indices (all data)	0.0411, 0.0799	0.0417, 0.0792	0.0554, 0.0910	0.0551, 0.0981
Max. peak/hole (e. Å^−3^)	0.290/−0.173	0.196/−0.178	0.460/−0.464	0.605/−0.379

## Data Availability

Crystallographic data for **1**–**4** have been deposited with the Cambridge Crystallographic Data Center as supplemental publication numbers CCDC 2178844, 2178845, 2178848, and 2178849, respectively. Copies of the data can be obtained free of charge via http://www.ccdc.cam.ac.uk (accessed on 30 August 2022).

## Supplementary Materials

The following supporting information can be downloaded at: https://www.mdpi.com/article/10.3390/molecules28114529/s1, Figure S1: The infrared spectra of complex **1**; Figure S2: The infrared spectra of complex **2**; Figure S3: The infrared spectra of complex **3**; Figure S4: The infrared spectra of complex **4**.

## Sample Availability

Samples of the compounds are available from the authors.
