# Supplementary material for: Synthesis, Structures, and Magnetism of Four One-Dimensional Complexes Using [Ni(CN)4]2− and Macrocyclic Metal Complexes
Source: Molecules. 2023 Jun 2;28(11):4529. doi: 10.3390/molecules28114529 (PMC10254484; doi:10.3390/molecules28114529)
Supplement: Supplementary file 1 [file molecules-28-04529-s001.zip › molecules-2359071-supplementary.pdf]

Supporting Information for

Synthesis, structures and magnetism of four one-dimensional materials using $[Ni(CN)_4]^{2-}$ and macrocyclic metal complexes

Guang-Chuan Ou*, Qiong Wang, Ying-Zhi Tan, Qiang Zhou and Fei Zeng

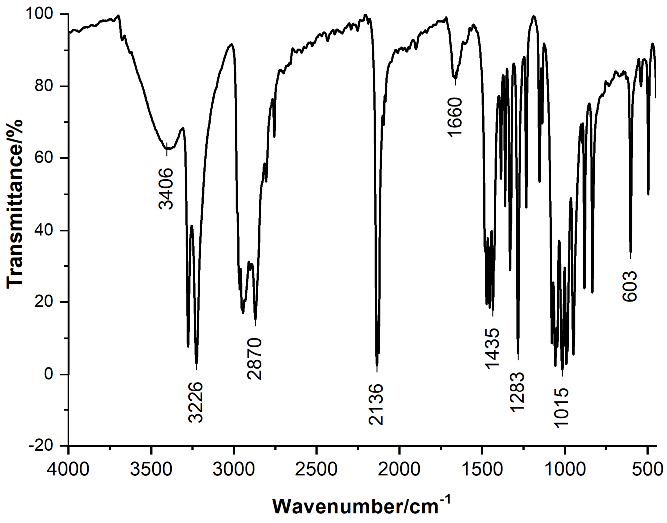

Figure S1. The infrared spectra of complex **1.**

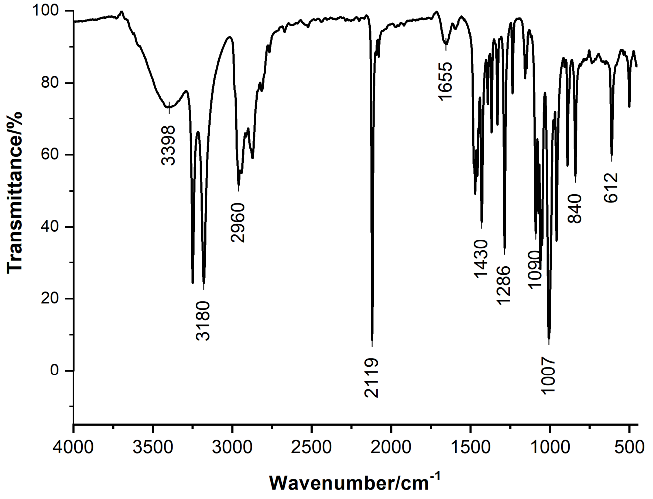

Figure S2. The infrared spectra of complex **2.**

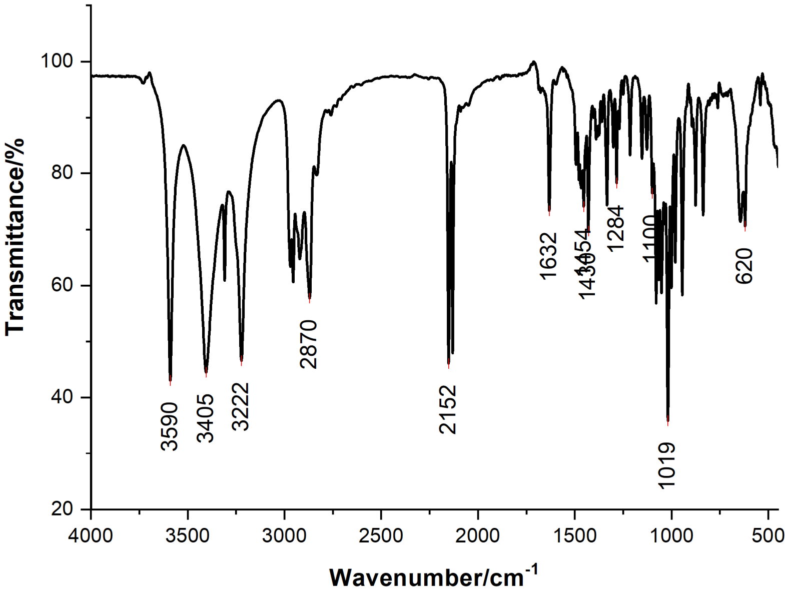

Figure S3. The infrared spectra of complex **3.**

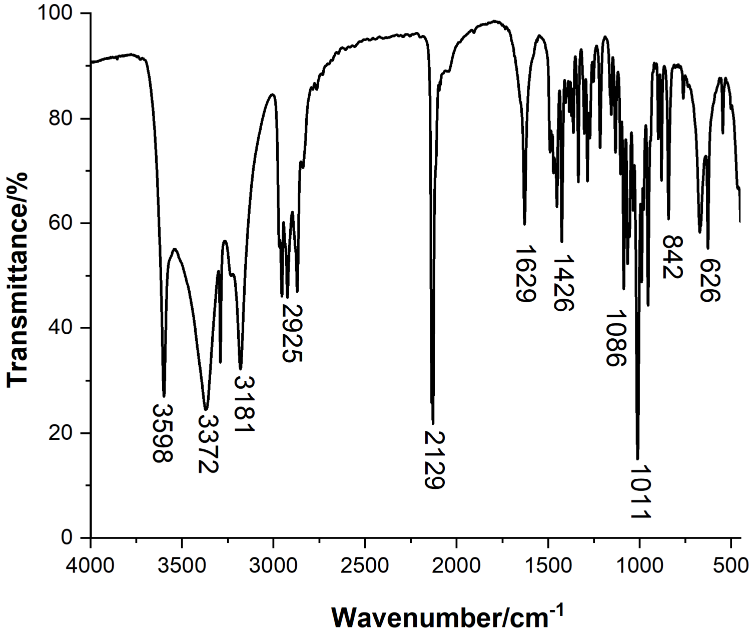

Figure S4. The infrared spectra of complex **4.**
